# Inhibition of Glycogen Synthase Kinase 3β Increases the Proportion and Suppressive Function of CD19^+^CD24^hi^CD27^+^ Breg Cells

**DOI:** 10.3389/fimmu.2020.603288

**Published:** 2020-12-04

**Authors:** Jinyang Li, Ji Gao, Haoming Zhou, Jinren Zhou, Zhenghua Deng, Yunjie Lu, Jianhua Rao, Guwei Ji, Jian Gu, Xinxiang Yang, Yongxiang Xia, Xuehao Wang

**Affiliations:** ^1^ Hepatobiliary Center, The First Affiliated Hospital of Nanjing Medical University, Nanjing, China; ^2^ Key Laboratory of Liver Transplantation, Chinese Academy of Medical Sciences, Nanjing, China; ^3^ NHC Key Laboratory of Living Donor Liver Transplantation, National Health Commission, Nanjing, China; ^4^ Hepatopancreatobiliary Surgery, The Third Affiliated Hospital of Soochow University, Changzhou, China

**Keywords:** liver transplantation, graft-versus-host disease, mBreg cells, GSK-3β, NFATc1

## Abstract

CD19^+^CD24^hi^CD27^+^ memory Breg cells exhibit decreased abundance in patients with chronic graft-versus-host disease (cGVHD) after liver transplantation and produce less IL-10 than those from patients without cGVHD and healthy donors. Due to the lack of Breg cells and the difficulty in expanding them *in vitro*, in mouse models and early human clinical trials, the adoptive transfer of Breg cells to autoimmune diseases is greatly restricted. Glycogen synthase kinase 3β (GSK-3β) is a multifunctional serine/threonine (ser/thr) protein kinase that can participate in B cell growth, metabolic activity, and proliferation. Phosphoprotein array analysis showed that p-GSK-3β-s9 was highly expressed in mBreg cells. Furthermore, here, we demonstrated that GSK-3β expression in mBreg cells is lower than that observed in B cells by flow cytometry. We found that the treatment of B cells with the specific GSK-3β inhibitor SB216763 can significantly increase the proportion and immunosuppressive function of mBreg cells *in vitro*. Nuclear factor of activated T cells (NFAT) is one of a pivotal regulator of gene expression in adaptive immune system. Here, we observed that inhibition of GSK-3β by SB216763 results in enhanced expression of NFATc1 in B cells, which is essential in regulating the ability of B cells to secrete IL-10. By constructing a xGVHD mouse model, we observed that SB216763-treated mBreg cells effectively prevent xenogeneic GVHD. Here we propose a novel strategy using SB216763 to inhibit GSK-3β and then enhance the proportion and immunosuppressive function of mBreg cells by increasing the expression of NFATc1. This approach may be used as a therapy to ameliorate GVHD and inflammatory diseases.

## Introduction

Graft rejection mediated by antibodies produced by B cells has been studied. Interestingly, recent researches have focused on the regulatory role of B cells, where B cells producing IL-10 to perform their immunosuppressive function, known as regulatory B cells (Breg cells), have been critical in the maintenance of graft tolerance (1). Various Breg subsets in humans, characterized by the expression of CD24^hi^CD27^+^or by CD24^hi^CD38^hi^, have been described a reduced function in specific autoimmune diseases (2), including experimental autoimmune encephalomyelitis (3), collagen-induced arthritis (4), and colitis (5). Our previous study showed that the acute rejection (AR) patients after liver transplantation showed a significant decreased proportion of CD19^+^CD24^hi^CD27^+^ memory Breg (mBreg) cells. After immunosuppressive treatment, the proportion of mBreg cells showed a significant increase (6). Recently, the role of Bregs in chronic graft-versus-host disease (cGVHD) has attracted increased attention (6). Although compelling evidence showed that human Breg cells can act as a regulator of autoimmune diseases (2), little is known regarding their role in GVHD, where CD4^+^CD25^+^ Tregs is the most watched (7–9). There have been precedents for *in vitro* amplification of Tregs and reinfusion for GVHD in patients with liver transplantation. Thus, the use of Bregs may be a new strategy for the treatment of GVHD in organ transplantation.

As a multifunctional serine/threonine (ser/thr) protein kinase, GSK-3β was originally identified as a main regulator in glycogen metabolism (10). Recent studies have uncovered that GSK-3β participates in the proliferation, differentiation, and Ig secretion of B cells (11). Furthermore, the results of our previous study showed that inhibiting GSK-3β in naïve T cells by SB216763 could enhance human iTreg differentiation and immunosuppressive function (12). Given that GSK-3β has been utilized as a marker for the diagnosis of GVHD (13), while no studies have confirmed that GSK-3β can modulate GVHD by regulating the differentiation and function of Breg cells. We believe this will be a new research direction.

As a vital regulator of gene expression in the adaptive immune system, nuclear factor of activated T cells (NFAT) has indispensable biological properties in human bodies. Reducing the expression of NFATs in the nucleus of Tregs will impair the differentiation of Tregs and inhibit the acquisition of the inhibitory phenotype, which is characterized by the secretion of anti-inflammatory cytokine IL-10 (14). In mice, the lack of NFATc1 and NFATc2 in T cells is related to the severely impaired production of a variety of cytokines (including IL-10, IL-2, IL-4, MCSF, IFN-γ, and TNF-α) (15). However, few studies have investigated the effect of NFAT on B cells. Thus, our objective was to determine whether NFAT is also involved in regulating mBreg differentiation and immunosuppression.

Utilizing the GSK-3β inhibitor SB216736, for the first time, we examined the role GSK-3β plays in the differentiation and suppressive function of CD19^+^CD24^hi^CD27^+^ memory Breg cells, both *in vitro* and *in vivo*. We also suggest a possible mechanism of how GSK-3β can modulate the differentiation and immunosuppression of mBregs by regulating the expression of NFATc1.

## Results

### Patients With Chronic GVHD Have a Decreased Proportion and Function of CD19^+^CD24^hi^CD27^+^ mBreg Cells After Liver Transplantation

**Figure 1:**
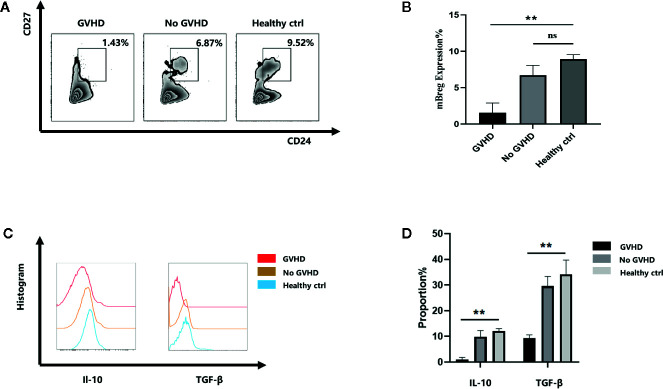
Patients with chronic GVHD have a decreased proportion and function of CD19^+^CD24^hi^CD27^+^ mBreg cells after liver transplantation (n = 5). Human peripheral blood samples were collected from 5 patients with GVHD, 5 patients without GVHD and 5 healthy volunteers **(A, B)**. The proportion of mBreg cells in the healthy control, GVHD patients, and no GVHD patients groups determined using flow cytometry **(C, D)**. Representative example of IL-10 and TGF-β expression by mBreg cells from patients with GVHD or without GVHD and healthy volunteers. The results are shown as the means ± standard error of the mean values (*P < 0.05 and **P < 0.01; ns, not signiﬁcant).

The results of our previous study demonstrated that acute rejection patients showed a remarkable decrease in the proportion of mBreg cells after liver transplantation (16). As mBreg cells participate in maintaining the immune balance after liver transplantation, we speculated that mBreg cells may participate in the occurrence of GVHD. First, we collected 5 samples of peripheral blood from patients after liver transplant when GVHD occurred (when a rash appeared and myelosuppression did not occur) to assess the proportion of mBreg cells. Flow cytometry results showed that the proportion of mBreg cells in patients with GVHD was significant lower than that observed in the healthy donors and patients without GVHD (**Figures 1A, B**). In addition to their direct contact-associated effects, Breg cells can also exert their immunosuppressive function by secreting different cytokines, among which TGF-β and IL-10 are the two vital cytokines (17). Therefore, we detected the secretion of TGF-β and IL-10 from mBreg cells. The results demonstrated that the secretion of IL-10 and TGF-β both decreased significantly compared with those from healthy donors and patients without GVHD (**Figures 1C, D**).

### GSK-3β Signaling Pathway Takes Part in the Differentiation of mBreg Cells

**Figure 2:**
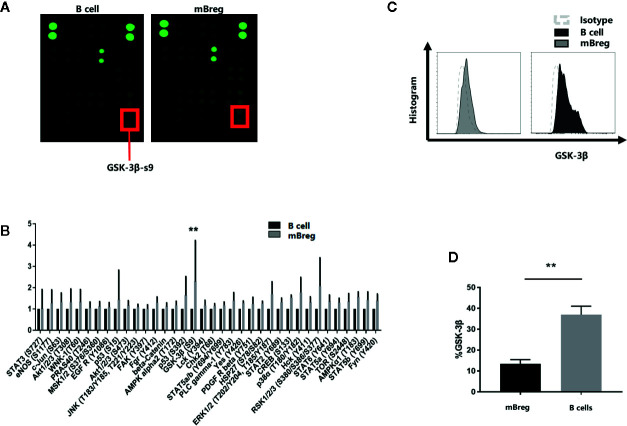
GSK-3β signaling pathway participates in the differentiation of mBreg cells (n = 3).. We collected the peripheral blood samples from the healthy volunteers. CD19^+^ B cells were sorted by CD19^+^ magnetic beads, while CD19^+^CD24^hi^CD27^+^ mBreg cells were sorted by Fluorescence Activated Cell Sorting **(A, B)**. Relative phosphorylation of GSK-3β-s9, determined using a phosphoprotein array **(C, D)**. The expression of GSK-3β in CD19^+^ B cells and memory Breg cells was detected by flow cytometry. The results are shown as the means ± standard error of the mean values (*P < 0.05).

To identify the signaling pathway involved in the differentiation of mBreg cells, CD19^+^ B cells and mBreg cells were sorted from the purified peripheral blood mononuclear cells (PBMC) of healthy volunteers by Fluorescence Activated Cell Sorting (FACS). A human phosphoprotein array was used to detect the changes in protein phosphorylation levels. The results showed that the phosphorylation of GSK-3β-serine 9 was increased significantly (**Figures 2A, B**), indicating that GSK-3β could be involved in regulating the differentiation of mBreg cells. To confirm the variations of the expression of GSK-3β among different B cell subsets, we used flow cytometry to evaluate the expression of GSK-3β total protein in CD19^+^ B cells and mBreg cells and observed that GSK-3β expression was significantly lower in mBreg cells (**Figures 2C, D**).

### Inhibiting GSK-3β in CD19^+^ B Cells Can Promote Breg Cell Differentiation

**Figure 3:**
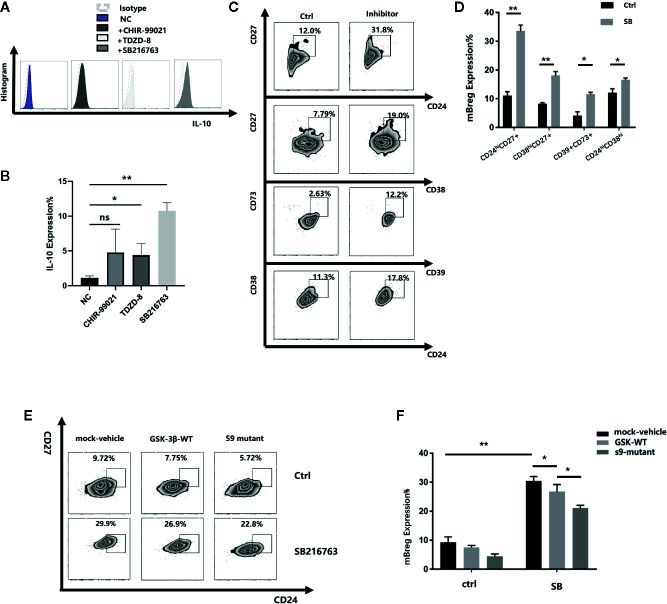
Inhibiting GSK-3β in CD19^+^ B cells can promote the differentiation of Breg cells (n = 3). CD19^+^ B cells were sorted by CD19^+^ magnetic-activated cell sorting from PBMC from healthy donors, which were stimulated with LPS, GSK-3β inhibitors were added to the medium **(A, B)**. IL-10 expression in B cells after adding 3 different inhibitors, SB216763, CHIR-99021, and TDZD-8 **(C, D)**. The proportion of transitional B cells (CD19^+^CD24^hi^CD38^hi^), mBreg cells (CD19^+^CD24^hi^CD27^+^), plasmablasts (CD19^+^CD27^hi^CD38^hi^), and CD19^+^CD39^+^CD73^+^ B cells after 48 h of coculture with or without SB216763 **(E, F)**. GSK-3β and GSK-3β-s9 site serine mutant lentiviruses were added with or without SB216763, the proportion of mBreg cells was detected using flow cytometry. The results are shown as the means ± standard error of the mean values (*P < 0.05 and **P < 0.01; ns, not signiﬁcant).

To determine the relationship between GSK-3β and Breg cell differentiation, we cocultured the purified CD19^+^ B cells with or without SB216763, CHIR-99021, and TDZD-8, three GSK-3β inhibitors. As IL-10 is one of the most recognized markers of Breg cells, we tested the proportion of CD19^+^IL-10^+^ Breg cells (B10 cell) and observed that all the three inhibitors could enhance the expression of IL10 compared with the negative control, and SB was the most effective. (**Figures 3A, B**). Therefore, we chose SB216763 in subsequent experiments. Because Breg cells do not have a defined phenotype, after 48 h of coculture, we selected seven of the most common Bregs to assess the changes in their proportion. Among these seven Bregs, the proportions of transitional B cells (CD19^+^CD24^hi^CD38^hi^), mBreg cells (CD19^+^CD24^hi^CD27^+^), plasmablasts (CD19^+^CD27^int^CD38^hi^), and CD19^+^CD39^+^CD73^+^ B cells increased significantly (**Figures 3C, D**). In contrast, no striking changes were observed in the other three Bregs (CD19^+^TIM1^+^ B cells, CD19^+^CD25^+^CD71^+^CD73^-^B cells, and CD19^+^CD28^+^CD1d^+^IgM^+^CD147^+^ B cells, data not shown). Of the 4 types of Bregs that showed remarkable changes, mBregs exhibited the largest changes in its proportions. We speculated that GSK-3β is crucial in the differentiation of mBreg cells. GSK-3β serine residues phosphorylation results in its loss of activity. At present, the phosphorylation of serine 9 is the most thoroughly studied (18). Young et al. (19). demonstrated that various GSK-3β inhibitors can significantly increase the levels of phospho-GSK-3β. To further verify the roles of GSK-3β and p-GSK-3β-s9 in the differentiation of mBreg cells, we subsequently assessed whether increased GSK3β activity affects mBreg differentiation. To this end, we infected a lentivirus expressing wild-type GSK3β to CD19^+^ B cells, and the results showed that GSK3β overexpression significantly reduced the proportion of mBregs compared with that observed in the control group. Using a lentivirus with an amino acid site mutation in the serine 9 position (the s9 site was disturbed), the proportion was further decreased (**Figures 3E, F**). The above results suggested that SB216763 can inhibit the activity of GSK-3β by increasing the phosphorylation of GSK-3β-s9 and then enhance the proportion of mBreg cells.

### Inhibiting GSK-3β With SB216763 Increases the Suppressive Activity of mBreg Cells

**Figure 4:**
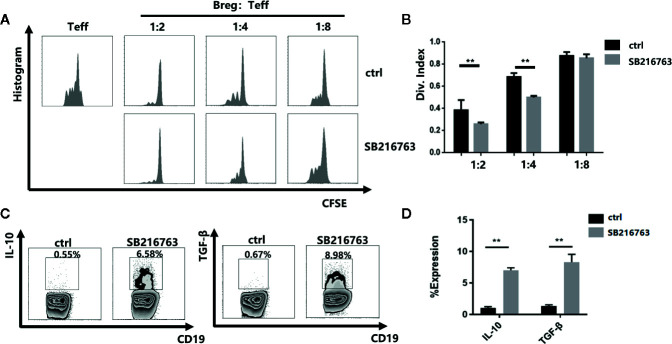
Inhibiting GSK-3β with SB216763 can enhance the inhibitory function of mBreg cells and affect the expression of related cytokines *in vitro* (n = 3). CD19^+^ B cells were sorted and cultured with SB216763 for 72 h with the stimulation of LPS **(A, B)**. Division index of CD8+ T cells mediated with anti-CD3 proliferation *Ex vivo*, ratio range 1:2 to 1:8 (B cells: PBMCs) was detected using CFSE dye dilution **(C, D)**. Representative example of IL-10 and TGF-β expression in B cells treated or not treated with SB216763. IL-10 and TGF-β increased significantly in B cells treated with SB216763 compared with that observed in the control. The results are shown as the means ± standard error of the mean values (**P < 0.01).

To further evaluate the effect of GSK-3β inhibition on the functional properties of mBreg cells, we utilized a carboxyfluorescein diacetate succinimidyl ester (CFSE)-based proliferation assay with CD19^+^ B cells to PBMC ratios ranging from 1:2 to 1:8 with or without SB216763 treatment. In the absence of SB216763, by detecting the proliferation of CD8+T cells, the inhibitory function of mBreg cells was significantly reduced compared with the mBreg cells added SB216763 (**Figures 4A, B**). Flow cytometry were utilized to test whether SB216763 could increase the production of IL-10 and TGF-β in mBreg cells. As expected, incubating CD19^+^ B cells with SB216763 could strikingly enhance the production of IL-10 and TGF-β compared to the untreated group (**Figures 4C, D**). These data demonstrated that inhibiting GSK-3β with SB216763 can enhance the inhibitory function of mBreg cells and affect the expression of related cytokines *in vitro*.

### Inhibition of GSK-3β With SB216763 Results in Enhanced NFATc1 Expression in mBreg Cells

**Figure 5:**
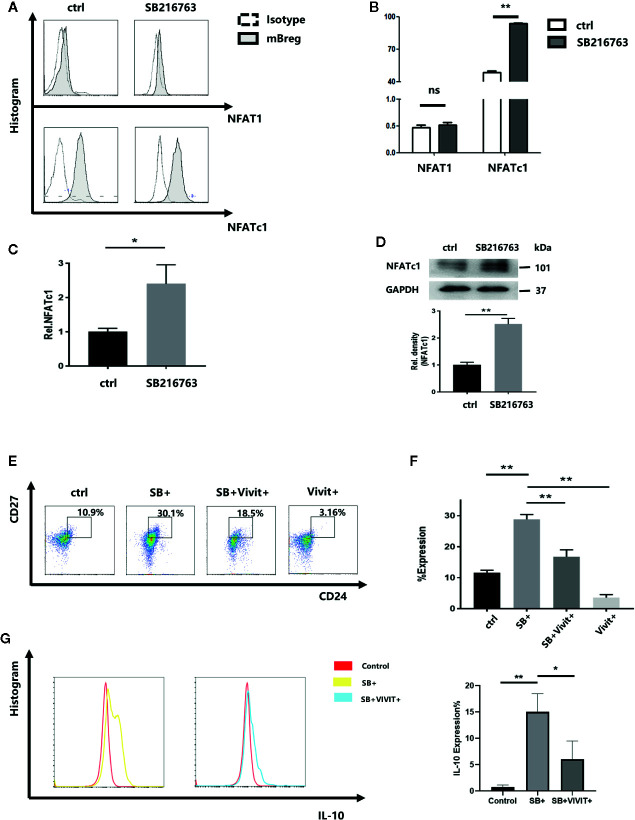
Treatment with SB216763 results in enhanced NFATc1 expression in mBreg cells (n = 3). Sorted CD19^+^ B cells were added with or without SB216763 for 72 h with the stimulation of LPS **(A, B)**. The expression of NFAT1 and NFATc1 of mBreg cells from different groups were detected by flow cytometry **(C, D)**. NFATc1 mRNA and protein expression in mBreg cells from each group, were detected by RT-qPCR and Western blotting, respectively **(E, F)**. The proportion of mBreg cells in the four groups (control, SB+, SB+VIVIT+, and VIVIT+) was measured using flow cytometry **(G)**. The proportion of IL10 in each group (control, SB+, SB+VIVIT+) by flow cytometry. The results are shown as the means ± standard error of the mean values (*P < 0.05 and **P < 0.01; ns, not signiﬁcant).

Chan R. Beals et al (20). demonstrated that GSK-3β can enhance the nuclear export of NF-ATc, which has been shown to involve the catalytic activity of GSK-3β. Therefore, the effect of GSK-3β inhibition by SB216763 on mBreg cells were assessed. Purified CD19^+^ B cells with LPS were cultured with or without SB216763. By using flow cytometry analysis, we assessed the untreated and SB216763-treated cultures for NFAT1 and NFATc1 levels while gating for the CD19^+^CD24^hi^CD27^+^ mBreg cells (**Figures 5A, B**). The results showed that NFAT1 expression in B cells and mBreg cells is lower than that of NFATc1. NFATc1 was significantly elevated in mBreg cells after treatment with SB216763, while NFAT1 showed no obvious changes. Therefore, we believed that SB216763 influenced the expression of NFATc1 rather than NFAT1. To further elucidate the impact of GSK-3β on NFATc1, we utilized RT-qPCR to assess the mRNA level of NFATc1, and the results were in line with our expectations (**Figure 5C**). In addition, NFATc1 protein expression was measured by Western blot analysis (**Figure 5D**). To further confirm the role of NFATc1 in the differentiation of mBreg cells, a specific NFATc1 inhibitor (21), VIVIT was added to the B cell culture system, which led to a significant reduction in the production of mBreg cells. In the presence of both SB216763 and VIVIT, the proportion of mBreg cells was significantly higher than that observed in the control group but remained significantly lower than the group treated with SB216763 alone (**Figures 5E, F**). In addition, after VIVIT was added, the proportion of IL10 decreased significantly compared with SB+ group (**Figure 5G**).

### Inhibition of GSK-3β by SB216763 Promotes the Suppressive Function of mBreg Cells *In Vivo*

**Figure 6:**
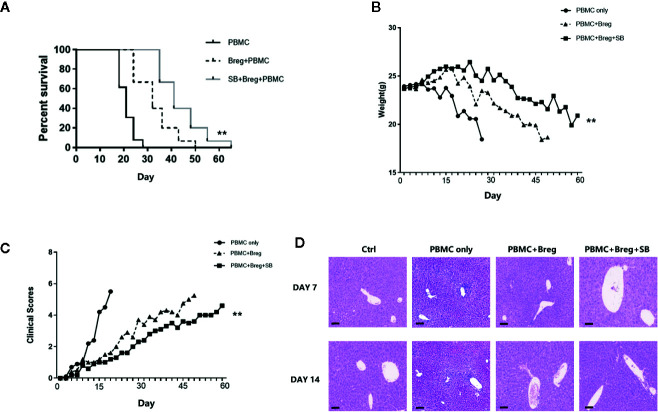
In the xenogeneic graft-versus-host disease (xGVHD) model, mBreg cells treated *in vitro* with SB216763 can protect organs from immune damage and reduce mortality (n = 5). Sorted human memory Breg cells by Fluorescence Activated Cell Sorting (FACS) from healthy volunteers were treated with or without SB216763 for 3 days. After the treatment, allogeneic PBMCs (10×10^6^) and mBreg cells (10×10^6^) were collected and transferred into NOD CRISPR Prkdc Il2r gamma (NCG) mice to test the immunosuppressive function of mBreg cells (± SB216763) in preventing GVHD. For the PBMC-only, PBMC+Breg, and PBMC+Breg+SB groups, n = 5, 5, and 5, respectively **(A)**. Mice used in experiments were injected with PBMC-only, PBMC+Breg, and PBMC+Breg+SB (**P < 0.01), Kaplan-Meier survival curves showed the results **(B)**. Average body weight of mice surviving on a given day in each group (**P < 0.01) **(C)**. Average clinical scores of GVHD in each group of mice surviving on the given day (**P < 0.01) **(D)**. We humanely sacrificed the NCG mice in the different groups on days 7 and 14 in another independent xGVHD experiment; Hematoxylin-Eosin (HE) staining was utilized for pathological examination of the liver in each group (n = 3 per group). The results shown represented two independent xGVHD experiments (*P < 0.05, bar = 100μm).

Because SB216763 could enhance mBreg cell differentiation and function *in vitro*, we next assessed whether SB216763-treated mBreg cells would be more effective at preventing xenogeneic GVHD. To this end, we constructed the same xGVHD mouse model that was used in our previous study (22, 23). *Ex vivo*-purified memory Breg cells were untreated or treated with SB216763 for 48 h and then were injected with allogeneic PBMCs (1:1 ratio; 10 × 10^6^) into the specific highly immunodeficient NSG mice. As shown in **Figure 6A**, both groups of mice receiving mBreg cells had significant reduced GVHD-induced lethality compared with that observed in the PBMC-only control group. Same results as *in vitro* experiments, compared with the PBMC-only and SB216763-untreated groups, the survival period of the SB216763 group mice was significantly prolonged, and the weight loss was significantly delayed (**Figure 6B**). The clinical indicators of GVHD in mice were also evaluated, weight loss, hair texture, skin integrity, posture, and activity were included. The results showed that compared with the control and untreated groups, the clinical symptoms of GVHD were fewer in SB-treated group (**Figure 6C**). We humanely killed mice in the four groups on days 7 and 14 after infusion, and the liver pathological changes were assessed by HE staining in another independent xGVHD experiment. As shown in **Figure 6D**, on the 7th day, the livers of mice in the PBMC-only group exhibited notable inflammatory cell infiltration in, while the SB216763-treated and -untreated groups had only mild inflammatory cell infiltration. On day 14, compared with the other groups, the inflammatory cell infiltration in the livers of mice in the SB-treated group was still mild.

## Discussion

B cells participate in the occurrence of autoimmunity and allogeneic immunity diseases by providing costimulation factors, antigens and cytokines to T cells (24). B cells possess regulatory properties, termed Bregs, are potent regulators of the immune response in different inflammatory settings (3, 4, 25–28). Evidence now suggests that regulatory B cells also play a significant role in organ transplantation (29–31). Previous studies have identified several Breg phenotypes. However, the phenotype of Breg cells has not yet been determined. Iwata et al. described a human equivalent of mBreg cells with a CD19^+^CD24^hi^CD27^+^ phenotype (32). In chronic GVHD, mBreg cells can provide protection by the suppressive effects and inhibit proliferation of effector T cells through IL‐10 secretion and cell‐to‐cell contact involving CTLA-4. Some studies have confirmed the effect of immunosuppressants on Bregs. In a transgenic model of amyotrophic lateral sclerosis, IL-10^+^ regulatory B cells adoptive transfer therapy could effectively reduce the number of bone marrow-derived macrophages in the central nervous system (33). Song et al (34). showed that Sirolimus could amplify regulatory B and T cells in liver transplant patients. In this study, we tested five patients with chronic GVHD after liver transplantation and observed that the proportion of mBreg cells decreased significantly in the GVHD acute phase compared to patients without GVHD and healthy patients. In addition, the mBreg cells from GVHD patients secreted reduced IL-10 and TGF-β. Although limited by the small number of cases, these results showed that mBreg cells could participate in the occurrence of GVHD and could be prevented or treated by increasing the proportion and function of Bregs in the blood of transplant patients. However, clinical trials and treatments using Bregs are confronted by multiple challenges: First of all, the number of Bregs in peripheral blood is too low to extract sufficient Bregs; furthermore, the *in vitro* amplification, survival and function of Bregs are still important issues. Graham et al (19). showed that the inhibition of GSK-3β potentiates Treg cell suppressive activity and that it does so by increasing Treg cell survivability. Therefore, we speculated that GSK-3β could be a possible new target for regulating the differentiation and immunosuppressive function of Bregs in immune diseases.

At present, there are still little studies about the role of GSK in lymphocytes, possibly due to the redundant functions of the alpha and beta subtypes. Jellusova et al (35). proved that the B cell intrinsic requirements of GSK-3 in humoral response, including the initiation and maintenance of the GC response. Furthermore, it also showed that GSK-3 activity can inhibit the accumulation of B cells, as well as the growth, proliferation, and metabolic activity of B cells. However, no studies have investigated the role of GSK-3β in the differentiation and function of Bregs. The successful clinical application of adoptive transfer Bregs must solve the problems of the limited number of active Bregs and their low survivability *in vitro*. We added three different GSK-3β inhibitors to B cells and found all the three inhibitors could enhance the expression of IL10 compared with the negative control, but SB216763 was the most effective. Our study is the first to reveal that the inhibition of GSK-3β by SB216763 can significantly promote the proportion and immunosuppressive function of mBreg cells by mediating NFATc1 expression. We showed that by utilizing the specific GSK-3β inhibitor SB216763 *in vitro*, the proportion of mBreg cells increased significantly. IL-10 and TGF-β are the primary cytokines responsible for the immunosuppressive function of Bregs (17). After allogeneic stem cell transplantation, compared with healthy donors and patients without cGVHD, chronic GVHD patients had fewer Bregs in peripheral blood and produced less IL-10 (6). Inhibiting GSK-3β can also enhance the expression and secretion of these two cytokines in mBreg cells, more than just increasing the quantity of mBreg cells. SB216763 has been widely used to study of the biological function of GSK-3β by increasing the phosphorylation of GSK-3β on serine 9. In this study, we constructed plasmids to overexpress wild-type or mutant (at the serine 9 position) GSK-3β. Compared to cells harboring the wild-type GSK-3β construct, the ratio of mBreg cells in cells harboring the mutant GSK-3β construct decreased. These results show that SB216763 could inhibit the activity of GSK-3β by altering the phosphorylation of GSK-3β-s9 and then increase the proportion of mBreg cells.

Previous studies has demonstrated the crucial role of calcineurin NFAT signaling pathway in regulating IL-10 production mediated by BCR (36), and deletion of IP_3_Rs leads to impairments in activated-NFAT expression, resulting in reduced numbers of IL-10-producing regulatory B cell. Overexpression of GSK-3β was shown to block the Ca^2+^-calcineurin-induced nuclear translocation of coexpressed NFATc in COS cells and could also inhibit transcription directed by endogenous NFAT or AP-1 components (20). In this study, we demonstrated that the inhibition of GSK-3β by SB216763 could enhance the expression of NFATc1. In the presence of SB216763, adding the specific NFAT inhibitor VIVIT could partially inhibit the effect of SB216763 on the differentiation and function of mBreg cells. Some properties of NFAT and NF-κB are similar, including rapid nuclear translocation upon antigenic stimulation and similar DNA binding domains. While NF-κB controls both innate and adaptive immune responses, NFAT controls the adaptive immune system (37). The role of NF-κB in the differentiation of mBreg cells remains to be investigated. In addition to NFAT, previous studies have shown that high levels of p-GSK-β-S9 lead to increased expression of β-catenin (38). GSK-3β plays an important role in regulating multiple signaling pathway, and because of the most important of these is Wnt/β-catenin signaling pathway (39, 40), we surmised that this signaling pathway may also be involved in these changes and will be a direction of our future research. Consistent with the *in vitro* results, inhibiting GSK-3β by SB216763 could also increase the proportion and suppressive function of mBreg cells *in vivo*, which is the core of adoptive therapy. We observed that inhibition of GSK-3β by SB216763 had better curative effect on GVHD, resulting in significant prolonged survival and an obvious delay in weight loss. In addition, compared with other groups, the SB-treated group had fewer clinical symptoms of GVHD. Subsequently, the pathological examinations including HE staining showed that GSK-3β knockdown can enhance the protective effect on GVHD.

It has been show that Treg adoptive cellular therapy has the ability to ameliorate autoimmune disease, graft rejection, and GVHD (41, 42). At present, the clinical use of Bregs is hindered by the challenges associated with the limited quantity of Bregs and their survival time being too short *in vitro*. Therefore, an optimal *in vitro* expansion protocol for Bregs is essential for Breg-based therapy. In summary, here, we demonstrated that a GSK-3β/NFATc1 pathway plays a crucial role in the differentiation and immunosuppressive function of mBreg cells both *in vitro* and *in vivo*. Thus, focusing on GSK-3β may represent a potentially new starting point in Breg clinical trials and treatments.

## Materals and Methods

### Patients

From February 2016 to December 2018, five patients with GVHD and five patients without GVHD in the Hepatobiliary Center, The First Affiliated Hospital of Nanjing Medical University received cardiogenic death (DCD) donor liver transplants are included in this study (**Table 1**). This study was conducted according to the recommendations of the Institutional Review Board of the First Affiliated Hospital of Nanjing Medical University, and all subjects’ written informed consent was obtained. All subjects submitted written informed consent in accordance with the Declaration of Helsinki. The research protocol got the approval from the Institutional Review Board of the First Affiliated Hospital of Nanjing Medical University (approval number 2015-SRFA-095).

**Table 1:** Patients and healthy volunteers demographics.

NO	Age	Sex	HBV	Blood Type	Primary Diagnosis	Initial IS Regiments
1	57	M	+	A	HCC	T+My+S
2	57	M	+	O	HCC	T+My+S
3	68	M	+	O	HCC	T+My+S
4	63	M	+	B	Liver cirrhosis	T+My+S
5	66	F	+	O	Liver cirrhosis	T+My+S
6	57	M	+	O	HCC	T+My+S
7	58	M	+	A	HCC	T+My+S
8	59	M	+	A	HCC	T+My+S
9	60	M	+	B	Liver cirrhosis	T+My+S
10	69	F	+	O	Liver cirrhosis	T+My+S
11	25	M	–	A	/	/
12	28	F	–	B	/	/
13	30	M	–	A	/	/
14	26	M	–	O	/	/
15	39	M	–	O	/	/

No. 1–5, patients with GVHD; No. 6–10, patients without GVHD; No. 11–15, Healthy volunteers; M, male; F, female; HCC, hepatocellular carcinoma; IS, immunosuppressive;

T, tacrolimus; My, mycophenolate mofetil; and S, steroids.

### Mice

Purchasing the highly immunodeficient NOD CRISPR Prkdc Il2rγ (NCG) mice from the Model Animal Research Center of Nanjing University and placed them in a facility free of specific pathogens. Each miniature isolation cage can accommodate up to 5 mice. All the mice were gender matching and age matching (6-8 weeks). Nanjing Medical University approved the Animal experiment protocols.

### Cell Puriﬁcation and Culture

Healthy donors PBMC seperation product came from the Department of Hematology, Jiangning Hospital, Nanjing Medical University. B cells (CD19+) was separated and purified from PBMC (Amersham Biosciences, Ficoll-Hypaque) by magnetic activated cell sorting (MACS) using the autoMACS Pro Separator instrument (Miltenyi Biotec, Germany) in accordance with the manufacturer’s protocol. CD19^+^CD24^hi^CD27^+^ mBreg cells were sorted from PBMCs by Fluorescence Activated Cell Sorting.

100 000 CD19^+^ B cells/well were cultured in RPMI-1640 medium, 25 mM HEPES and ultraglutamine (Switzerland, Lonza, Basel) were added to the medium. Providing 10% FBS and 100 ng/mL lipopolysaccharide (LPS) to the medium on a 96-well plate at 37°C in an incubator under a humidified atmosphere with 5% CO_2_ for 48 h with or without stimulation. The CD19^+^ B cells were cultured with 5 μM SB216763 (Sigma-Aldrich) or with 0.05% DMSO (vehicle control) in the presence or absence of 10 μM VIVIT for 48 h. Plasmid transfection was performed with Lipofectamine 2000 (Waltham, MA, USA, Invitrogen, ThermoScientic).

### Antibodies and Flow Cytometry

The following human-speciﬁc antibodies were purchased and used for ﬂow detection, including against CD4(PE-CY7), CD27(APC), CD19(APC-CY7), CD1d(APC), IgM(FITC), CD24(PerCP/CY5.5), CD147(PE), CD25(FITC), CD71(PE), CD73(PerCP/CY5.5), CD39(FITC), TIM-1(PE), NFAT1(PE), and NFATc1(FITC), which were purchased from BioLegend, and GSK-3β(BV421), which was purchased from BD Pharmingen. Antibodies against IL-10(PE), TGF-β(FITC), GSK-3β(BV421), NFAT1(PE), and NFATc1(FITC) were used for intracellular flow cytometry assays, while the others were used for cell surface staining. Using CATON II instrument (BD Bioscience) for sufficient sample acquisition, using FlowJo VII (TreeStar) to analyze the data.

### Western Blot Analysis and Antibodies

Incubating the cells with specific inhibitors for 60 min before switching the cells to NaCl solution. After treatment, ice-cold PBS was used to wash the cells once, and then adjusted to the osmotic pressure by adding NaCl. In order to measure the expression of NFATc1 protein, samples were collected in a solution containing 150 mM NaCl, 1% Triton X-100 and 50 mM Tris-HCl (pH 7.4). Before use, adding protease inhibitor tablets (Roche) to all the buffers. Finally, the samples were analyzed by Western blot and quantify by infrared imaging (Odyssey, Li-Cor).

### Quantitative Real Time Polymerase Chain Reaction (RT-qPCR)

Using an EasyPure RNA Mini kit to collect RNA from the cell samples (Beijing, China; TransGen Biotech). Using TransScrip First-Strand cDNA Synthesis SuperMix to synthesis Complementary DNA (cDNA) (Beijing, China; TransGen Biotech), following the manufacturer’s instructions. Using GAPDH as a control gene, and the relative gene expression of NFATc1 (from Ribobio Corporation, Guangzhou, China) determined using an Applied Biosystems 7500 Real-Time PCR System Fast SYBR Green Master Mix (#4385612) and Assay on Demand primer/probe kits (Waltham, MA, USA, Applied Biosystems). Analyzing the results to acquire the average delta CT. We followed the following conditions to conduct PCR experiment: 94°C, 5s, 40 cycles; 60°C, 30s; 95°C incubation for 10 min.

### Lentivirus Production and Transduction

293T cells (6×10^6^ cells/100 mm plate) were transiently transfected with recombinant lentivirus, and 3 plasmids were co-transfected with calcium phosphate precipitation (1): pCMVΔR8.91 (2), pCDNA3.1-3xFlag-GSK3β (3), pLR-VSV-G (CA, USA, Invitrogen Carlsbad), a plasmid expressing the vesicular stomatitis virus envelope glycoprotein (G), at 20:15:6 μg/plate. Collecting the supernatants, and then 4°C ultra-high speed centrifugation, 26,000 rpm, 2 h, 500-fold concentration later, -80°C frozen storage for use. Virus titers were determined by quantitative reverse transcription PCR (RT-qPCR).

### Phosphoprotein Array Analysis

CD19^+^ B and CD19^+^CD24^hi^CD27^+^ mBreg cells were separated from PBMCs. SPRINGBIO 720 protein microarray technology was used to assay four pairs of samples in a multistep process involving protein extraction, BCA protein quantification, protein labeling, and biotin-labeled protein sample hybridization with the chip. A GenePix 4000B instrument was used for image scanning and excitation at 532 nm. Using the fluorescence scanner to scan the chip and then determine the degree of phosphorylation according to the fluorescence intensity. GenixPro 6.0 was used to analyze the data.

### Xenogeneic GVHD Model and Pathological Examination

Each micro-isolator cage houses up to 5 NCG mice 6 to 8 weeks old in a special pathogen-free facility. Irradiating the mice with 150 cGys on day 0. Then, human PBMCs (10×10^6^) were washed and transferred with or without mBreg cells (10×10^6^) added. The clinical scores based on the GVHD symptoms of the mice were recorded daily. Weighing each of the experimental mouse every other day. An independent xGVHD model experiment was performed as described above. Sacrificing the mice on days 7 and 14 and then made them into liver paraffin specimens. Performing Hematoxylin-eosin (HE) staining on paraffin specimens (thickness: 4mm) to detect the degree of inflammatory and tissue damage.

### Suppression Assays

As mentioned earlier, the inhibition test carboxyfluorescein succinimidyl ester (CFSE) was used to test the inhibitory function of mBreg cells cultured *in vitro* (43). Brieﬂy, labeling the puriﬁed PBMCs with CFSE (Invitrogen) and then cocultured with CD19^+^ B cells (1:2 to 1:8 CD19^+^ B cells/PBMCs). Cells were collected and incubated with the specific antibodies against CD8 four days later. Division Index was used to explain the suppression (FlowJo, TreeStar).

### Statistical Analysis

Using SDS v2.3 to analyze RT-qPCR data, while the survival data were analyzed using Prism 5 (Mantel-Cox). Analyzing all the other data by analysis of variance or Student’s t-test. Probability (P) values ≤ 0.05 were considered signiﬁcant.

## Data Availability Statement

The raw data supporting the conclusions of this article will be made available by the authors, without undue reservation.

## Ethics Statement

The studies involving human participants were reviewed and approved by Ethics Committee of the First Affiliated Hospital of Nanjing Medical University. The patients/participants provided their written informed consent to participate in this study. The animal study was reviewed and approved by Institutional Animal Care and Research Advisory Committee of Nanjing Medical University. Written informed consent was obtained from the individual(s) for the publication of any potentially identifiable images or data included in this article.

## Author Contributions

JL and JGao designed this study, performed the core experiments, processed the experiment data, and wrote the first draft. HZ, JZ, ZD, and YL performed part of the experiments. JR and GJ provided part of the experiment data. JGu discussed and interpreted the data. JGu discussed and interpreted the data; XY provided guidance on experimental design. YX and XW provided ideas for the article, corrected design errors, and wrote the manuscript. All authors contributed to the article and approved the submitted version.

## Funding

This work was supported by grants from the National Natural Science Foundation of China (No. 81771716, No. 31930020, No. 81870488, No. 81521004, No. 81530048), the Key Talent’s Subsidy Project in “Science and Education Strong Health Project of Jiangsu Province,” and Key Laboratory of Liver Transplantation, Chinese Academy of Medical Sciences (2018PT31043, 2019PT320015).

## Conflict of Interest

The authors declare that the research was conducted in the absence of any commercial or financial relationships that could be construed as a potential conflict of interest.
